# A Case of Pyoderma Gangrenosum on the Lip

**DOI:** 10.1002/ccr3.70462

**Published:** 2025-04-20

**Authors:** Mari Nakanishi, Makoto Kondo, Keiichi Yamanaka

**Affiliations:** ^1^ Department of Dermatology Mie University, Graduate School of Medicine Tsu Japan; ^2^ Department of Dermatology Yokkaichi Municipal Hospital Yokkaichi Japan

**Keywords:** fosfomycin, lip, pathergy, prednisolone, pyoderma gangrenosum, ulcer

## Abstract

Pyoderma gangrenosum should be considered in the differential diagnosis of ulcerative lip lesions in children. Long‐term management may require low‐dose oral steroids. In cases where flares are triggered by infection, intermittent antibiotic therapy can be beneficial.

**Abbreviations**
*C. acnes*
: *Cutibacterium acnes*
PG: pyoderma gangrenosum

Pyoderma gangrenosum (PG) is an inflammatory disease characterized by severe pain and rapidly progressive skin ulceration [1, 2]. The most common site of onset is the lower limbs, but it sometimes occurs on the head and face, especially in children. PG is often associated with underlying systemic disease and can also be exacerbated by minor trauma, a condition known as pathergy. The current report describes a case of PG that developed on the lip of a 10‐year‐old boy after a minor trauma.

The 10‐year‐old male lightly injured his lower lip 6 days before his initial visit, and the following day, his lips swelled. He was prescribed Cefcapene pivoxil and olopatadine by his local doctor, but his condition did not improve, and he was referred to our hospital. At the initial examination, the entire lower lip was swollen, and the left lower lip showed ulcer formation with severe pain (Figure 1). A biopsy was performed on the swollen lower lip skin ulcer to differentiate hereditary angioedema and granulomatous cheilitis, revealing inflammatory cell infiltration without granuloma formation. No systemic symptoms or other clinical or skin findings ruled out Behcet's disease. Immunostaining of the specimen revealed negativity for cytomegalovirus, Epstein–Barr virus, herpes simplex viruses 1 and 2, and fungi. The dental metal for orthodontic purposes was removed, but the patient still had an ulcer and swelling of the lip. PCR sequence analysis using *Cutibacterium acnes* (*C. acnes*)‐specific primer showed positive results from the bottom of the biopsy specimen, and sequence data was compatible with that of *C. acnes*. Then, oral fosfomycin was started. Considering the diagnostic criteria of PG of the lip [3], the diagnosis of PG was made, and the topical application of Difluprednate ointment, and then daily 5 mg of oral prednisolone was supplemented. Three months later, the swelling and ulceration significantly improved (Figure 2). It has now been 10 months since the start of treatment, and he is currently taking 5 mg of oral steroids and continuing fosfomycin on weekends. He has a mild clinical flare‐up on the withdrawal of antibiotics and requires a period of maintenance therapy.

**FIGURE 1:**
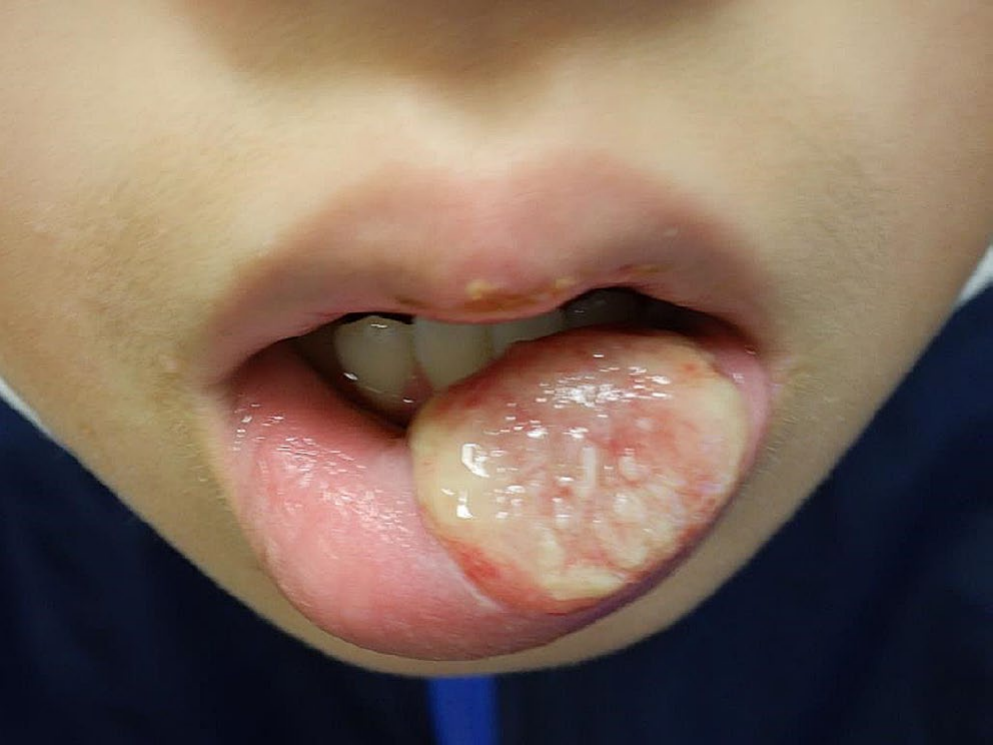
On initial examination, the patient presented with a swollen and intensely painful ulcer formation on the lower lip.

**FIGURE 2:**
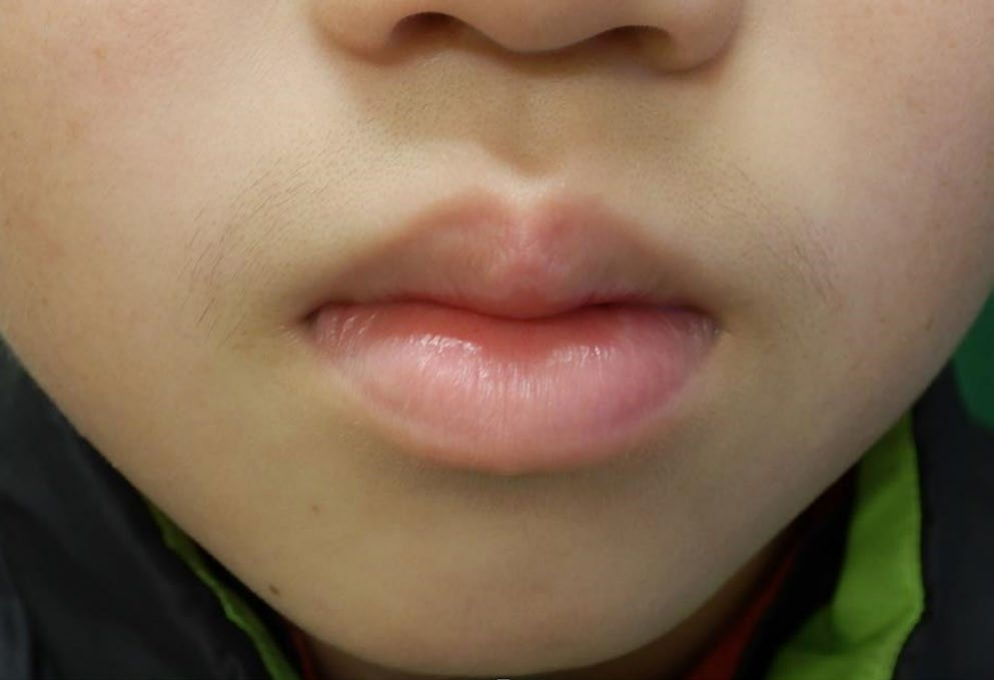
After the introduction of oral and topical steroids, the ulcer and swelling improved significantly.

The triggers of PG in the current case are unknown. Although the result of the blood examination was negative for rheumatoid factor and antinuclear antibodies, there is a family history of rheumatoid arthritis. A metal plate worn for orthodontic purposes may also have induced minor trauma. *C. acnes* was detected deep in the biopsy specimen. The possibility of detecting commensal bacteria cannot be ruled out, but it is relevant in that the patient also responded to antibiotic therapy. This case may require long‐term oral steroids, as there is a tendency for mild clinical flare‐ups without weekend antibiotics.

## Author Contributions

**Mari Nakanishi:** conceptualization, data curation, project administration. **Makoto Kondo:** conceptualization, investigation, writing – review and editing. **Keiichi Yamanaka:** conceptualization, data curation, investigation, project administration, supervision, writing – original draft, writing – review and editing.

## Ethics Statement

The research was conducted in accordance with the Declaration of Helsinki. The patient gave us consent for his photographs and medical information to be published in print and online with the understanding that this information is publicly available. The paper is exempt from ethical committee approval because of the single case study.

## Consent

Written consent for publication was obtained from the patient.

## Conflicts of Interest

The authors declare no conflicts of interest.

## Data Availability

The data that support the findings of this study are available on request from the corresponding author. The data are not publicly available due to privacy or ethical restrictions.
